# Supplementary material for: Propidium Monoazide (PMAxx)-Recombinase Polymerase Amplification Exo (RPA Exo) Assay for Rapid Detection of Burkholderia cepacia Complex in Chlorhexidine Gluconate (CHX) and Benzalkonium Chloride (BZK) Solutions
Source: Microorganisms. 2023 May 26;11(6):1401. doi: 10.3390/microorganisms11061401 (PMC10302078; doi:10.3390/microorganisms11061401)
Supplement: Supplementary file 1 [file microorganisms-11-01401-s001.zip › microorganisms-2362136-supplementary.pdf]

SUPPLEMENTARY MATERIAL

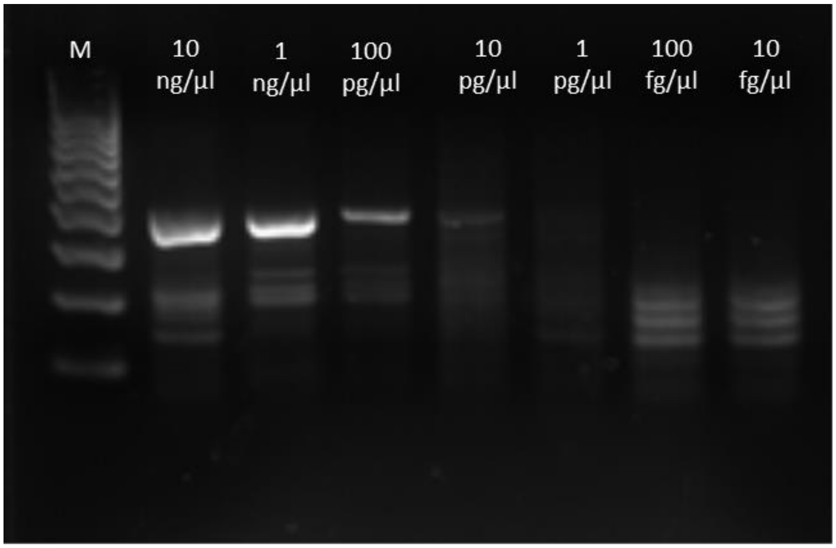

Figure S1: Sensitivity analysis of *B. cenocepacia* J2315 by PMAxx-RPA exo assay with agarose gel electrophoresis. DNA samples (94.3 ng/µl) were spectrophotometrically determined at A260 and using 10-fold serial diluted genomic DNA (approx. 10 ng/µL, 1 ng/µL, 100 pg/µL, 10 pg/µL, 1 pg/µL, and 100 fg/µL) of *B. cenocepacia* J2315.

Table S1. Sequence information of the cluster 3558

<table>
<tr><th>No. of cluster</th><th>ID</th><th>Size</th><th>Start</th><th>Stop</th><th>Sequence</th><th>Consensus sequence</th></tr>
<tr><td rowspan="3">3558</td><td>1</td><td>26</td><td>283</td><td>309</td><td>ACGCTGTCGTCGACGATCATCAGCCT</td><td rowspan="3">ATGTCTGAAATXATTCCXCTCGGXXXXTGGGTCGACCXXXXCGTXCAXTAXXTXCTXGA
XCAXGAXGCXXXXACXTTCGAXXCGXTXGGXCXXGCGATCGAXXGXCTXGCXGCXXTX
XTCGAXCAXXGXCTXCAGGCXXTXCCGATGTGGXTGXTGATGGCXXTCTTCATCGGXXT
XGGXXTGTGGCGXGTXGGCTGGCGXTTXGCXXTGTTXXXXACXXXXTCXXTXCTXCTGA
TXTTXGCXACXGGXTTCTGGGAXCAGACXXTXATXACGCTXGGXCTXACGCTGTCGTC
GACGATCATCAGCCTXGXGCTXGGCATXCCGCTCGGCXTCTGGGCXGCXAAGAGCAAX
XXGGTXGCXGCXXXXXTGCGXCCGXTXCTXGAXXTGATGCAXACGATGCCXGCXTTCGT
XTAXCTGATTCCGGCXGCXATGXTGTTCGGXCTXGGXCGXGTGCCXGGXATCCTGTCGA
CGGTGATCTTCGCGATGCCGCCXGCXGTXCGXCTXACGAGCCTXGGXATXCGXCAXG
TGAAXCGXGAGATXGTCGAXGCXGGXCAGGCXTTXGGXTGXACGCCXTGGCAXXTXCT
XTACAAXGTXCAXTTXCCGAAXGCGXTGCCGTCGATCATGCAXGGXGTXAACCAGACG
ATCATGATGGCGCTGTCGATGGTXATCATXGCXTCGATGGTXGGXGCXGGCGGXCTXG
GCAACGAXGTGCTXGCXAGXATCCAGCGXCTXGAXATCGGXCTXGGXTTCGAXAGCGG
XXTGTCGGTXGTGXTGCTXGCGATCATXCTXGAXCGXATCACCGAXAGCTTXGGXCGXG
CXCCXGGXXCXGXXXXXGCXCCGXTXTTCXCXGGXCTXAAGCAXXTXXTXCGXXXXAA
XXXXXXXXXCXXXCAXGCXTXA</td></tr>
<tr><td>2</td><td>32</td><td>460</td><td>492</td><td>ATCCTGTCGACGGTGATCTTCGCGATGCCGCC</td></tr>
<tr><td>3</td><td>32</td><td>634</td><td>666</td><td>AACCAGACGATCATGATGGCGCTGTCGATGGT</td></tr>
</table>
